# Cumulative DNA damage by repeated low-dose cisplatin injection promotes the transition of acute to chronic kidney injury in mice

**DOI:** 10.1038/s41598-021-00392-6

**Published:** 2021-10-22

**Authors:** Noriyuki Yamashita, Kunihiro Nakai, Tomohiro Nakata, Itaru Nakamura, Yuhei Kirita, Satoaki Matoba, Benjamin D. Humphreys, Keiichi Tamagaki, Tetsuro Kusaba

**Affiliations:** 1 Department of Nephrology, Graduate School of Medical Science, Kyoto Prefectural University of Medicine, 465 Kajii-cho, Kamigyo-ku, Kyoto, 602-8566 Japan; 2 Department of Cardiovascular Medicine, Graduate School of Medical Science, Kyoto Prefectural University of Medicine, Kyoto, Japan; 3 Institute of Experimental Medicine and Systems Biology, RWTH Aachen University, Aachen, Germany; 4 Division of Nephrology, Washington University School of Medicine in St. Louis, St. Louis, USA

**Keywords:** Acute kidney injury, Renal fibrosis

## Abstract

Cisplatin is a commonly used anticancer drug, but nephrotoxicity is a dose-limiting adverse effect. Recent experimental and clinical observations have demonstrated that multiple injections of cisplatin induce the transition to chronic kidney disease; however, the underlying mechanisms remain unclear. We found that multiple injections of higher doses of cisplatin in a shorter interval affected the severity of kidney injury, causing kidney fibrosis to develop at a later time point. An additional injection of cisplatin during the recovery period after a prior injury, when proximal tubule epithelia are actively proliferating, induced substantial tubular injury by inducing more severe DNA damage than that induced by a single injection. Lineage tracing analysis of proximal tubular epithelia demonstrated that the tubular epithelia that underwent multiple rounds of cell division after multiple injections of cisplatin existed at the chronic phase, and these populations often expressed vcam1 + , suggesting the induction of proinflammatory failed-repair tubular epithelia. Our study revealed that as cisplatin exerts cytotoxic effects on actively proliferating cells, additional cisplatin injections before the completion of tubular repair exacerbates kidney injury through cumulative DNA damage. Appropriate both the setting of dosage and dosing intervals, with careful monitoring, are essential to prevent nephrotoxicity of repeated cisplatin treatment in cancer patients.

## Introduction

The clinical diagnoses of acute kidney injury (AKI) and chronic kidney disease (CKD), have recently become considered as closely interconnected syndromes^1^. AKI is a risk factor for the development of future CKD, CKD is also a risk factor for AKI, and each are one of the largest risk factors for cardiovascular disease and mortality^2–4^. However, despite high cost and poor outcomes^3^, specifically targeted treatments are lacking. AKI has long been considered a reversible syndrome, but recent studies demonstrated AKI can increase the risk for CKD and end-stage renal disease^2,5^. Although the cellular and molecular mechanisms underlying this AKI to CKD transition are not completely understood, injury-induced maladaptive tubular repair, endothelial dysfunction, and sustained inflammation have been reported to accelerate further kidney damage, resulting in interstitial fibrosis and CKD^6–11^. The severity of AKI is a strong determinant for future CKD^12,13^. In addition, the frequency of AKI episodes is associated with the cumulative risk of developing CKD^5–7,14^. Recovery from AKI is often incomplete, and even in cases of milder AKI, there may be subclinical kidney damage not reflected in the serum creatinine concentration^5,6^.

Numerous insults, including those due to anticancer drugs and hemodynamic abnormalities cause AKI^15^. In patients with malignancy, anticancer drug-induced AKI is a serious complication because it restricts the next chemotherapy cycle and forces a switch to potentially less effective drugs, worsening prognosis. Cisplatin is one of the most frequently used platinum-based anticancer drugs for the treatment of many types of cancers, but the major dose-limiting side effect is nephrotoxicity, which occurs in approximately one-third of patients receiving cisplatin^16^. Cisplatin is absorbed through organic cation transporters (OCTs) expressed in renal proximal tubules, mainly at the basolateral side^17,18^. Accumulated cisplatin binds to DNA and causes defective DNA templates, and arrest of DNA synthesis and replication, resulting in subsequent DNA damage^16^. Although milder DNA damage can be repaired, severe DNA damage leads to irreversible injury and cell death^19^. The majority of apoptotic cells are observed in proximal tubules^20,21^, thus renal proximal tubular DNA damage is one of the most important mechanisms of cisplatin-induced nephrotoxicity.

In rodents, single high-dose cisplatin-induced AKI (≥ 20 mg/kg) is a well-established model of nephrotoxicity, but the high lethality rate precludes assessment of the AKI to CKD transition^22^. Furthermore, this model cannot accurately mimic the AKI to CKD transition observed in clinical practice^23^. Recently, a more clinically relevant mouse model, repeated low-dose cisplatin-induced kidney injury model, was established. In 2016, several studies investigated the effects of repeated low-dose cisplatin treatment, and they revealed that inflammation, maladaptive tubular repair, fibrosis, and loss of renal function developed after multiple episodes of milder AKI^22,24^. Of note, even a smaller single dose that did not cause apparent kidney damage caused prolonged renal damage and fibrosis with increased inflammation when repeatedly administered four times^22^. These studies opened up a new research area of repeated cisplatin-induced nephrotoxicity and several studies followed thereafter^25–27^. Although these mice models are well designed, the underlying mechanisms of this frequency-dependent kidney injury remain unclear. In particular, little is known about why a lower dose of cisplatin, which causes only mild reparable renal damage after a single dose, causes severe irreparable renal damage after multiple doses. Due to the lack of definitive treatments for cisplatin nephropathy, the prevention of kidney injury is the top priority; therefore, understanding the pathobiology is critical.

In general, after kidney injury and tubular cell death, neighboring dedifferentiated surviving cells play a key role by proliferative expansion with migration, which is followed by redifferentiation into mature tubular epithelia^28–31^. We hypothesized that cisplatin, an anticancer drug that generally causes DNA damage and cell death in rapidly proliferating cells, exacerbates DNA damage in regenerative proliferating renal proximal tubules after multiple rounds of cisplatin-induced injury and repair, thereby inducing extensive tubular damage and further maladaptive repair.

In the present study, we elucidated the mechanism of cumulative kidney damage by repeated cisplatin-induced kidney injury using mice models administered different frequencies, doses, and intervals of cisplatin. Repeated administration of cisplatin at shorter intervals or at a higher dose when tubular epithelia were undergoing repair and entering the cell cycle worsened the kidney damage by exacerbating DNA damage. Moreover, by lineage tracing analysis using specific reporter mice, we further confirmed that proximal tubular epithelial cells that were actively proliferating after injury led to tubular maladaptive repair in the later phase.

## Methods

### Animal experiments

Male C57BL/6 wild type mice were purchased from Shimizu, Inc (Kyoto, Japan). The cisplatin injury model was induced by intraperitoneal injection of cisplatin (Nippon Kayaku, Tokyo, Japan) in saline at a concentration of 6 mg/kg, 8 mg/kg, or 10 mg/kg body weight into male mice at the age of 8–10 weeks. Cisplatin was administered in the morning. Mice were anesthetized with isoflurane and then euthanized 3, 7, 14, or 28 days after the last cisplatin injection. Littermate control mice were used for comparison among groups.

For lineage tracing analysis, we recently generated C57BL/6 strain mice with the CreERT2 cassette in the SLC34a1 locus, which enables expression of Cre recombinase in the proximal tubules after tamoxifen injection^29^. SLC34a1GCE mice were crossed with the C57BL/6 strain R26tdTomato reporter mice, in which tdTomato is expressed after Cre-mediated recombination of the floxed stop cassette to obtain bigenic offspring. For genetic labeling, tamoxifen (Sigma Aldrich Co., LCC., St. Louis, MO) was dissolved in 3% (vol/vol) ethanol containing corn oil (Sigma Aldrich Co.) at a concentration of 10 mg/mL. Tamoxifen was injected intraperitoneally at the indicated dose either once or every other day.

All experiments were approved by the Experimental Animals Committee, Kyoto Prefectural University of Medicine, and were performed in accordance with the institutional guidelines, Guidelines for Proper Conduct of Animal Experiments by the Science Council of Japan, and the ARRIVE guidelines.

### Tissue preparation and histology

Mice were anesthetized and sacrificed, and kidneys were removed at the indicated time points. For frozen sections, kidneys were fixed with 4% paraformaldehyde (Wako Pure Chemical Industries, Ltd., Osaka, Japan) for 1 h on ice, incubated in 30% (vol/vol) sucrose in PBS at 4 °C overnight, embedded in optimum cutting temperature compound (Sakura FineTek Japan co., Ltd., Tokyo, Japan), and cut into 7-μm sections. For paraffin sections, the kidneys were fixed with 4% paraformaldehyde and embedded in paraffin by Applied Medical Research Laboratory (Osaka, Japan). Paraffin-embedded tissues were cut into 4-μm sections. PAS and Masson’s trichrome staining was performed according to standard procedures. The kidney histology was examined on formalin sections stained with PAS and Masson’s trichrome. The degree of interstitial fibrosis or tubular injury was scored semi-quantitatively in five of 25 consecutive non-overlapping cortical fields of kidney sections stained with PAS and Masson’s trichrome under high magnification. Interstitial fibrosis was quantified using the following scores: 0, 0%; 1, 1–10%; 2, 11–25%; 3, 26–50%; 4, 51–75%; and 5, 76–100%. Tubular injury was judged by tubular atrophy, tubular dilation, protein casts, necrotic cells, and brush border loss^31^.

### Immunofluorescence analysis

Immunofluorescent staining was performed according to previously described method^31^. Sections were rehydrated and permeabilized with 0.5% Triton X-100 in PBS for 5 min. Samples were blocked with 10% normal goat serum in PBS and sequentially incubated with the primary antibodies shown in Supplementary Table S1 for 1 h, followed by an incubation with dye-conjugate secondary antibodies (Supplementary Table S1) for 1 h. Nuclear counterstaining was performed using DAPI or DRAQ5 (DR50050; BioStatus, Leicestershire, UK; 1:2000), followed by mounting in Prolong-Gold (Thermo Fisher Scientific, Waltham, MA).

The quantification of tdTomato + , PDGFRβ + , LTL + , or vcam-1 + tubules was performed by measuring five high-power field (HPF) images of each kidney section in randomly selected cortical fields (n = 3). For clonal analysis, quantification of the number of consecutive tdTomato + cells in more than ten HPF images of each kidney section in randomly selected cortical fields were measured (n = 3–4).

### Immunohistochemistry

Immunohistochemistry staining was performed according to previously described method^31^. After deparaffinization, the sections were placed in citrate-buffered solution (pH 6.0) and boiled for 5 min to retrieve antigens. Endogenous peroxidase was quenched with 3.0% hydrogen peroxide in methanol for 20 min. Blocking was performed using 3.0% bovine serum albumin (BSA, Nacalai Tesque, Kyoto, Japan) in phosphate-buffered saline (PBS) for 30 min. Then, the sections were incubated with the primary antibodies shown in Supplementary Table S1, followed by an incubation with HRP-conjugated secondary antibodies (Supplementary Table S1). Diaminobenzidine (DAB) chromogenic substrate (K3468, Agilent Technologies, Inc., Santa Clara, CA) was used for color visualization, followed by counterstaining with hematoxylin.

### RNA extraction and real-time quantitative PCR

According to previously described method^31^, total RNA was extracted from the kidneys using TRIzol (Life Technologies, Inc., Carlsbad, CA) and Direct-zol™ RNA MiniPrep (Zymo Research Corporation., Irvine, CA). Two hundred nanograms of total RNA was reverse transcribed to synthesize cDNA using a PrimeScript RT reagent kit with gDNA Eraser (Takara Bio Inc., Shiga, Japan). The real-time detection of PCR products was performed using KAPA SYBR FAST qPCR Master Mix (2 ×) Universal (Kapa Biosystems, Wilmington, MA) and a Thermal Cycler Dice Real Time System (Takara Bio Inc.). All reactions were performed in duplicate. The primers for targets are listed in Supplementary Table S2.

### Comet assay

The comet assay was performed using the Comet Assay Kit (ab238544, Abcam plc., Cambridge, UK) according to the manufacturer’s protocol and previously described method^21^. In brief, mouse kidneys were removed and minced in a small amount of ice-cold PBS containing 20 mM EDTA. After removing the large pieces of tissue, the supernatant was passed through a 35-μm cell strainer. After centrifugation, the pellet was suspended at 1 × 10^5^ cells/ml in ice-cold PBS. Cell samples were mixed with comet agarose in a 1/10 ratio (v/v) and immediately transferred onto the slide glasses covered with comet agarose base layer. After incubating with pre-chilled lysis buffer, the slides were subjected to electrophoresis. Electrophoresis was performed in the Alkaline Electrophoresis Solution for the alkaline comet assay and in the TBE Electrophoresis Solution for the neutral comet assay. After electrophoresis, the slides were incubated with Vista Green DNA dye. Images were obtained by epifluorescence microscopy (IX71; Olympus, Tokyo, Japan) using the FITC filter. Ten pictures (5–15 cells per picture) were randomly taken, and the tail moment (tail length x tail % DNA/100) of more than 50 cells per group was calculated using Comet Score analysis software (TriTek Corp.).

### Statistics

Results are expressed as the mean ± standard error (SE). Each experiment was performed using at least three mice per group. Quantification was performed using at least five high-power field images for each kidney. For the clonal analysis, Quantification was performed using at least 10 high-power field images for each kidney. Statistical analysis was performed by the unpaired t-test for comparison of two variables and by analysis of variance and Tukey’s post hoc test for comparison of multiple variables. P-values < 0.05 were considered significant.

## Results

### Dose-dependent progression to CKD by repeated cisplatin administration

**Figure 1:**
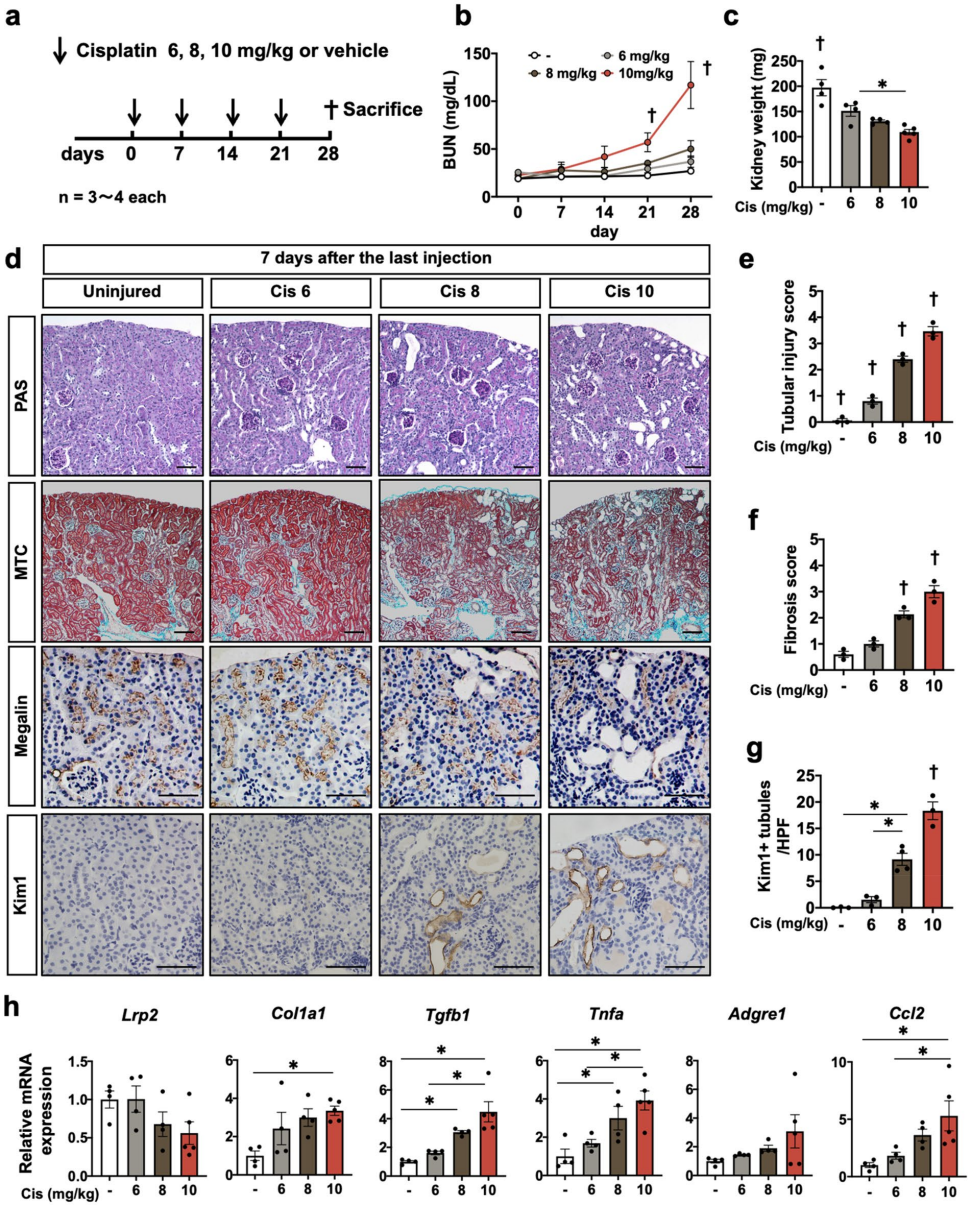
Dose-dependent response to repeated cisplatin induction. (**a**) Experimental scheme. Cisplatin was injected repeatedly at a different dose. (**b**) Dose-dependent BUN increase in repeated cisplatin-induced nephropathy. (**c**) Dose-dependent kidney weight reduction in repeated cisplatin-induced nephropathy. (**d**) Histological analysis of the kidney. PAS staining, Masson’s trichrome, and immunostaining of megalin and kim-1. (**e**, **f**, **g**) Quantification of the tubular injury score, fibrosis score, and the number of kim-1 + tubules. (**h**) Quantitative PCR of whole kidneys for the representative markers of mature tubules (*Lrp2*), fibrosis (*Col1a1 and Tgfb1*), and inflammation (*Tnfa, Adgre1*, and *Ccl2*). Data are the mean ± SE. *P < 0.05, †*P* < 0.05 vs other groups. Scale bar = 100 μm.

In order to evaluate the kidney injury after repeated injection of different doses of cisplatin in vivo, male wild type mice were administered 6, 8, or 10 mg/kg of cisplatin or saline intraperitoneally once a week for four weeks (Fig. 1a). After four injections of different doses of cisplatin in one-week intervals, the BUN significantly increased only in the mice receiving repeated cisplatin injection at 10 mg/kg (Fig. 1b). The average kidney weight was the smallest in the mice with repeated injection of 10 mg/kg of cisplatin (Fig. 1c). PAS and Masson’s trichrome staining revealed marked tubular damage and interstitial fibrosis in the kidneys of mice receiving 10 mg/kg of cisplatin, whereas those receiving 6 mg/kg exhibited slight tubular damage and interstitial fibrosis (Fig. 1d,e,f). Immunostaining of megalin, a marker of mature tubular epithelia, and of kim-1, a marker of tubular injury, revealed a large number of megalin-negative or kim-1-positive injured tubules in the kidneys of the mice receiving 10 mg/kg of cisplatin, whereas injured tubules were scarce in the kidneys of the mice receiving 6 mg/kg (Fig. 1d,g). Consistent with the pathological findings, downregulation of the *Lrp2* gene encoding megalin, upregulation of *Col1a1* and *Tgfb1* genes, markers of fibrosis, and upregulation of the *Tnfa gene, Adgre1* gene encoding F4/80, *and Ccl2* gene markers of inflammation, were noted by quantitative PCR in the kidneys of mice receiving 10 mg/kg of cisplatin (Fig. 1h).

### Seven-day interval after 10 mg/kg of cisplatin is insufficient for complete recovery

**Figure 2:**
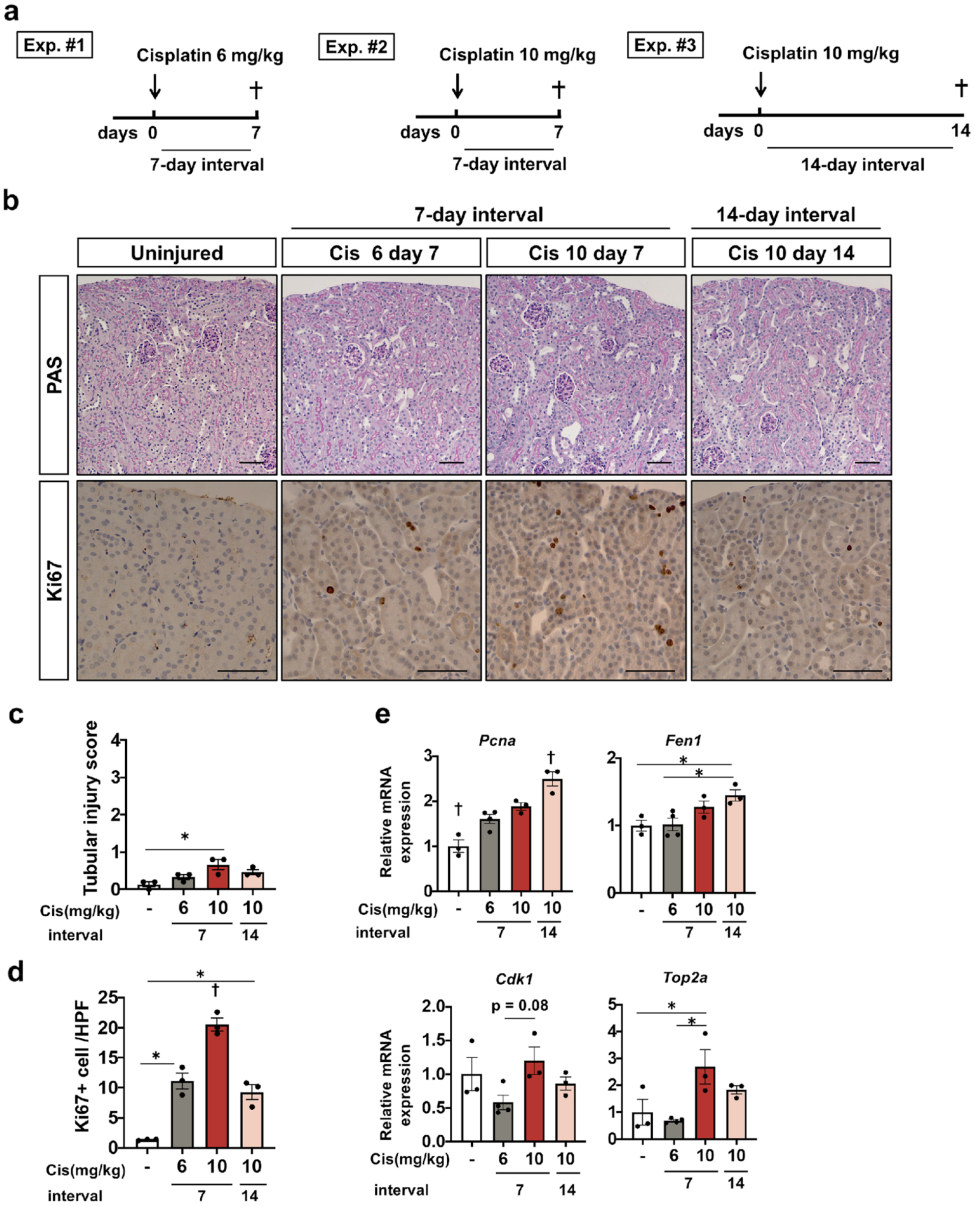
Assessment of repair and cell cycle state at seven or 14 days after a single cisplatin injection at a dose of 6 or 10 mg/kg. (**a**) Experimental scheme. A single dose of 6 mg/kg or 10 mg/kg of cisplatin was injected. The kidneys were assessed at seven or 14 days after the injection. (**b**) Histological analysis of the kidney. PAS staining, and immunostaining of Ki-67. (**c**, **d**) Quantification of the tubular injury score and the number of Ki-67 + tubules. (**e**) Quantitative PCR of whole kidneys for the cell cycle markers (*Pcna, Fen1*, *CDK1,* and *Top2a*). Data are the mean ± SE. *P < 0.05, †*P* < 0.05 vs other groups. Scale bar = 100 μm.

As repeated injections of 6 mg/kg cisplatin in a one-week interval did not cause apparent kidney injury and those of 10 mg/kg cisplatin accelerated kidney injury without causing lethality, we compared the difference in histological phenotypes at seven days after the first injection of 6 mg/kg or 10 mg/kg cisplatin (Fig. 2a). We also evaluated the mice at 14 days after a higher dose of cisplatin administration (Fig. 2a). PAS staining revealed no significant difference in renal histology among all experimental groups (Fig. 2b,c). According to quantitative PCR, the expression of the *Lrp2* gene, a marker of mature tubular epithelia, was preserved in all groups of mice receiving cisplatin. Although *Tnfa, Adgre1*, and *Ccl2* genes, markers of inflammation, and Acta2 gene encoding αSMA, marker of myofibroblast were slightly up-regulated in all groups, no significant different among cisplatin groups was observed (Supplementary Fig. S1). However, immunostaining of Ki-67, a marker of cells entering the cell cycle, revealed Ki-67 + tubular epithelial cells in the mice administered cisplatin, and the number of Ki-67 + epithelia was highest in the kidneys at seven days after 10 mg/kg cisplatin injection (Fig. 2b,d). We evaluated cell cycle-related gene expression by quantitative PCR. Consistent with Ki-67 staining, cell cycle markers (*Pcna, Fen1, Cdk1*, and *Top2a* gene) slightly increased in all groups receiving cisplatin (Fig. 2e). In particular, the expression of regulatory molecules associated with G2 /M phase, *Cdk1*, and *Top2a* increased in the kidneys at seven days after 10 mg/kg cisplatin injection (Fig. 2e). Taken together, although histological findings, and markers of mature tubular epithelia, inflammation, and myofibroblast activation did not markedly differ among the groups receiving cisplatin at day seven or at day 14, a larger number of tubular epithelial cells were still entering the cell cycle at day seven after a single injection of 10 mg/kg of cisplatin. Proximal tubular epithelia of kidneys at seven days after 6 mg/kg of cisplatin injection and at 14 days after 10 mg/kg of cisplatin injection had almost recovered, whereas those at seven days after 10 mg/kg of cisplatin injection were still entering the cell cycle, suggesting that they were undergoing repair.

### Two doses of 10 mg/kg cisplatin injection seven days apart caused the most extensive kidney damage through cumulative DNA damage

In order to elucidate the responsible mechanisms for the repeated cisplatin-induced AKI to CKD transition, we analyzed the phenotypes at the acute phase three days after different frequencies, doses, and intervals of each cisplatin injection.

**Figure 3:**
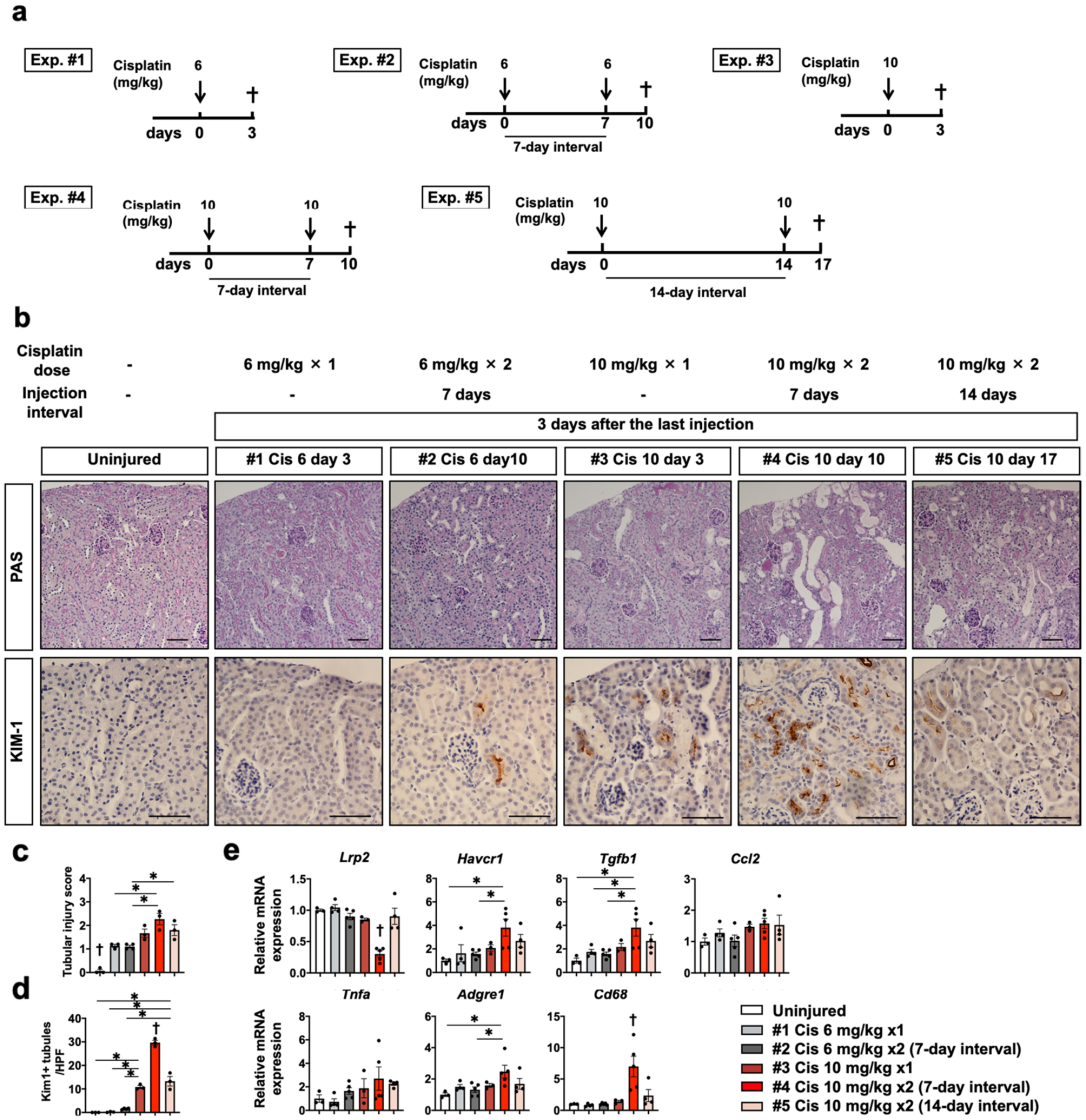
Assessment of kidney injury after different frequencies, doses, and intervals of cisplatin injection. (**a**) Experimental scheme. A single or double dose of 6 or 10 mg/kg of cisplatin was injected at the indicated time point. The kidneys were assessed at three days after the last injection. (**b**) Histological analysis of the kidney. PAS staining and immunostaining of kim-1. (**c**, **d**). Quantification of the tubular injury score and the number of kim-1 + tubules. (**e**) Quantitative PCR of whole kidneys for the representative markers of mature tubules (*Lrp2*), tubular injury (*Havcr1*) profibrotic signal (*TGFb1*), and inflammation (*Tnfa, Adgre1, Cd68,* and *Ccl2*). Data are the mean ± SE. *P < 0.05, †*P* < 0.05 vs other groups. Scale bar = 100 μm.

As a seven-day interval after 6 mg/kg cisplatin or 14-day interval after 10 mg/kg cisplatin injection is sufficient for recovery, but a seven-day interval after 10 mg/kg is not, we administered one dose of 6 or 10 mg/kg cisplatin, or two doses of 6 or 10 mg/kg cisplatin seven or 14 days apart to mice (Fig. 3a).

PAS staining and Immunostaining of kim-1 revealed extensive tubular injury in all groups receiving cisplatin, and injury was the most severe in mice receiving two doses of 10 mg/kg cisplatin seven days apart (Fig. 3b,c,d). In this group, downregulation of the *Lrp2* gene, a marker of mature tubular epithelia, upregulation of the *Havcr1* gene, a marker of tubular injury, upregulation of the *Tgfb1* gene, a profibrotic marker, and upregulation of *Tnfa*, *Adgre1*, *Cd68* genes, markers of inflammation, were marked based on quantitative PCR (Fig. 3e). Quantitative PCR also revealed that *Ccl2* gene was slightly up-regulated in all groups receiving cisplatin, however no significant different among groups was observed (Fig. 3e).

**Figure 4:**
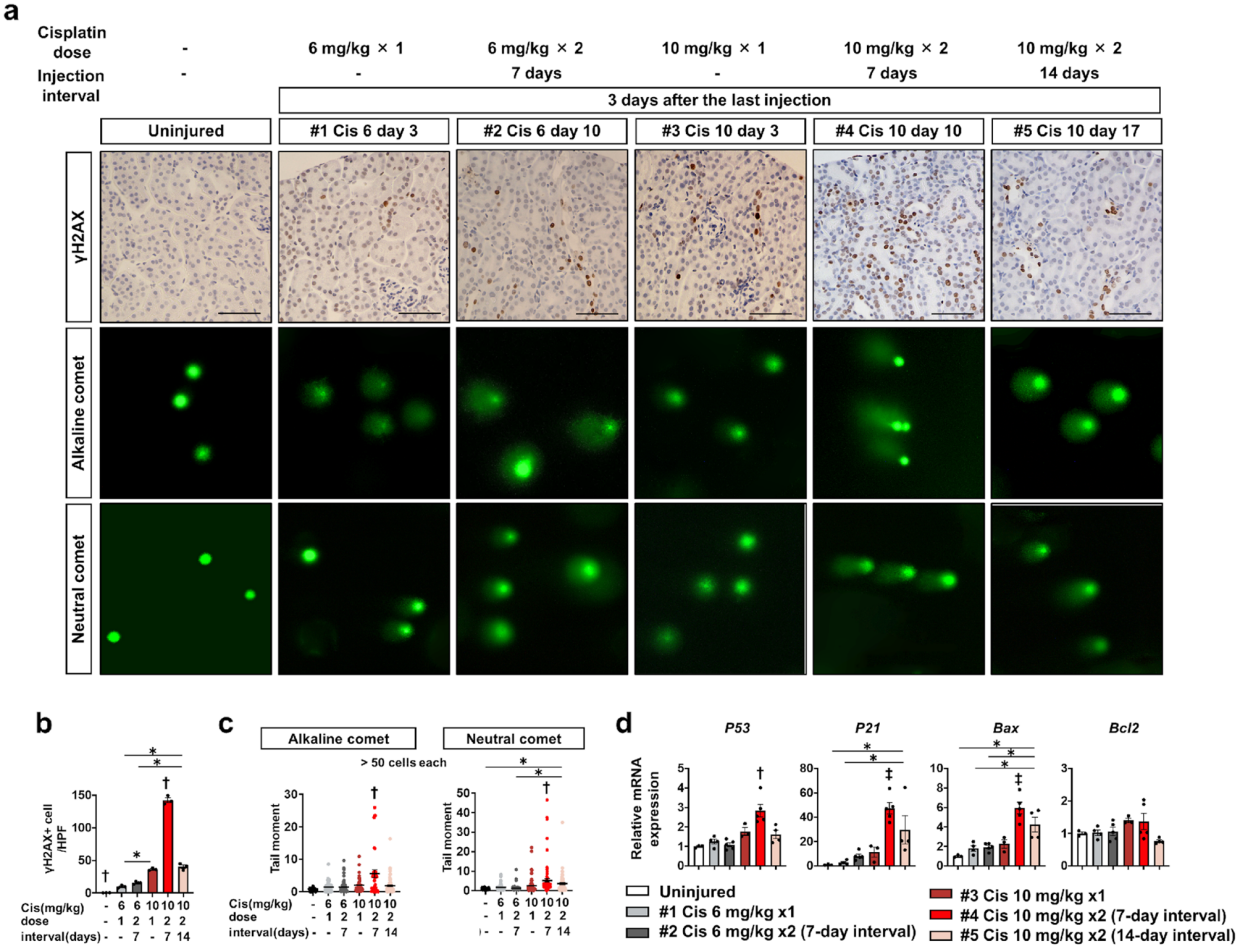
Assessment of DNA damage after different frequencies, doses, and intervals of cisplatin injection. (**a**) Representative images of immunostaining of γH2AX and comet assay of isolated kidney cells at three days after the last injection. (**b**) Quantification of the number of γH2AX + cells. (**c**) Quantitative analysis of alkaline comet and neutral comet. (**d**) Quantitative PCR of whole kidneys for the downstream target genes of DNA damage (*p53, p21*, *Bax,* and *Bcl2*). Data are the mean ± SE. *P < 0.05, †*P* < 0.05 vs other groups, ‡*P* < 0.05 vs Uninjured or #1–3 groups. Scale bar = 100 μm.

As cisplatin causes more severe DNA damage in rapidly proliferating cells, we hypothesized that additional cisplatin injection at seven days after injection of 10 mg/kg of cisplatin when a larger number of tubular epithelial cells were undergoing replication caused extensive DNA damage in tubular epithelia. We next performed immunostaining for γH2AX, a marker of DNA damage. A small number of γH2AX-positive tubular epithelial cells were observed in mice receiving one dose of 6 mg/kg cisplatin, and the number of those did not change significantly when they received two doses seven days apart (Fig. 4a,b). On the other hand, the number of γH2AX-positive tubular epithelial cells was higher in mice receiving one dose of 10 mg/kg cisplatin than in mice receiving 6 mg/kg, and it increased even more in mice receiving two doses of 10 mg/kg cisplatin seven days apart (Fig. 4a,b). Notably, even with the same two doses of 10 mg/kg cisplatin, the number of γH2AX was lower in mice receiving two dose of 10 mg/kg cisplatin 14 days apart than in mice receiving seven days apart, and this number was about the same as a single dose of cisplatin (Fig. 4a,b).

To quantify DNA damage in detail, we performed comet assay of isolated kidney cells. The comet tail moment in the alkaline condition (alkaline comet) reflects both DNA double-strand breaks (DSB) and single-strand breaks (SSB), whereas that in the neutral condition (neutral comet) reflects only DSB^32^. Results of comet assay showed the same trend of DNA damage as Immunostaining of γH2AX in each group. Both alkaline and neutral comet tail moments increased in kidney cells of mice receiving cisplatin and were further increased in those receiving two doses of 10 mg/kg cisplatin seven days apart (Fig. 4a,c). Upregulation of *p53, p21, Bax*, and *Bcl2* genes, downstream signals of DNA damage, in this group was noted by quantitative PCR (Fig. 4d). Quantitative PCR also demonstrated markedly high DNA repair-related gene expression in this group (Supplementary Fig. S2). This suggested that additional cisplatin injection before tubular repair is completed exacerbates kidney injury through extensive DNA damage, which can lead to chronic and irreversible kidney disease after repeated cisplatin-induced kidney injury.

### Repeated administration of 10 mg/kg cisplatin induced chronic and irreversible kidney disease along with tubular maladaptive repair, and fully differentiated cells are responsible for this repair process

**Figure 5:**
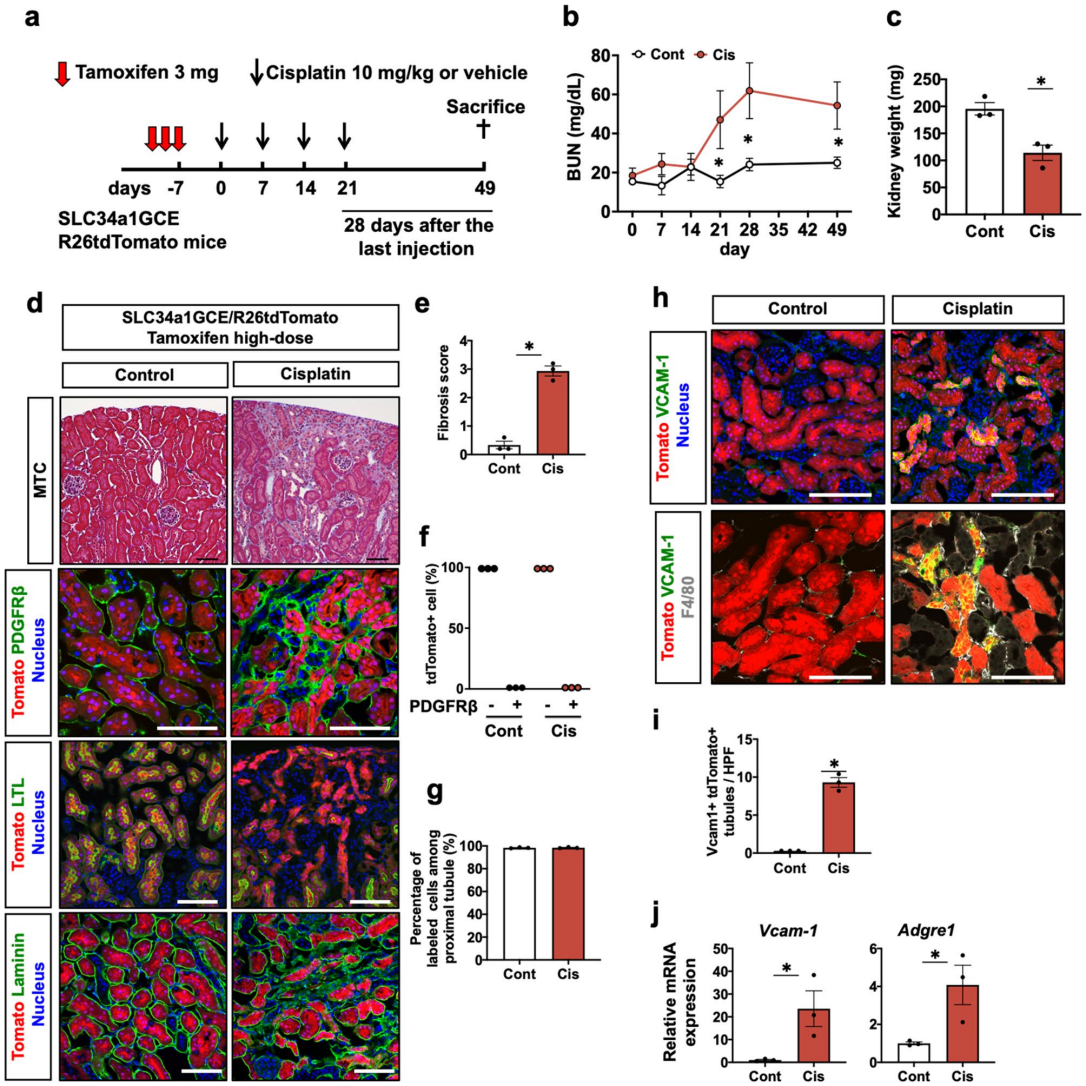
Maladaptive tubular repair and interstitial fibrosis after repeated administration of 10 mg/kg of cisplatin. (**a**) Experimental scheme. Repeated administration of 10 mg/kg of cisplatin to the SLC34a1GCE R26tdTomato mice after labeling of proximal tubular epithelial cells by multiple injections of high-dose tamoxifen. The kidneys were assessed at a later phase, 28 days after the last injection. (**b**) Chages in BUN level during and after repeated administrations of cisplatin. (**c**) The average kidney weight in the cisplatin and control groups. (**d**) Histological analysis of the kidneys in the cisplatin and control groups. Masson’s trichrome and immunostaining of PDGFRβ, LTL, and laminin. (**e**) Quantification of the fibrosis score. (**f**) Quantification of PDGFRβ positivity in the tdTomato + cells. tdTomato + tubules never merged with PDGFRβ. (**g**) Quantification of labeled cells. Labeled cells were not diluted in the cisplatin model. (**h**) Immunostaining of vcam-1 and F4/80. (**i**) Quantification of vcam-1 + tdTomato + tubules. (**j**) Quantitative PCR of whole kidneys for vcam-1 and adgre1 genes. Data are the average ± SE. *P < 0.05. Scale bar = 100 μm in MTC and 50 μm in others.

Lastly, we investigated the consequences of repeated cisplatin injury by lineage tracing analysis of proximal tubules using bigenic mice carrying a proximal tubule-specific tamoxifen-inducible Cre (SLC34a1GCE) and the tdTomato reporter (R26tdTomato)^29^. After exclusively labeling terminally differentiated proximal tubular epithelia by high-dose tamoxifen injection, the mice were administered 10 mg/kg of cisplatin or saline intraperitoneally once a week for four weeks and euthanized 28 days after the last cisplatin injection (Fig. 5a). BUN levels in mice receiving cisplatin gradually increased according to rounds of cisplatin injection and were still maintained 28 days after the last injection (Fig. 5b). The average kidney weight in mice receiving cisplatin was smaller than that in mice receiving saline (Fig. 5c). Masson’s trichrome and immunostaining of PDGFRβ revealed extensive interstitial fibrosis in the cisplatin model (Fig. 5d,e). Immunostaining of LTL, a healthy tubule marker, demonstrated numerous LTL- tdTomato + tubules, reflecting maladaptive repair in the cisplatin model. In addition, they were not co-stained with PDGFRβ, suggesting that tdTomato + tubular epithelia do not transdifferentiate into interstitial fibroblasts (Fig. 5d,f), which is consistent with our previous reports using the IRI model^29,31^. Furthermore, there was no dilution of tdTomato labeling among proximal tubules (Fig. 5d,g), suggesting that terminally differentiated tdTomato + tubular epithelia were solely responsible for the tubular repair in this model. Downregulation of the *Lrp2* gene denoting mature tubular epithelia, and upregulation of the *Havcr1* gene denoting tubular injury, *Ccl2* gene indicating inflammation, and *Col1a1* and *Tgfb1* genes indicating fibrosis were noted in the chronic phase after repeated cisplatin injection (Supplementary Fig. S3).

Recent single-nucleus RNA sequencing of a mouse model of AKI identified distinct proinflammatory vcam1 + proximal tubules that fail to repair after ischemia reperfusion injury (IRI)^33^. Vcam1 + failed-repair proximal tubules were also observed in our mouse model and they were surrounded by F4/80 + macrophages, whereas they were scarce in the control kidneys (Fig. 5h,i, and Supplementary Fig. S4). Consistent with the immunofluorescence study, upregulation of *vcam1* and *adgre1* genes encoding F4/80 was noted in the cisplatin model by quantitative PCR (Fig. 5j). Taken together, repeated cisplatin-induced kidney injury leads to chronic and irreversible kidney disease accompanying proinflammatory failed-repair proximal tubules and fibrosis.

### Clonal analysis revealed that cell cycle entry and proliferation of tubules during repeated injury lead to maladaptive repair in the chronic phase

**Figure 6:**
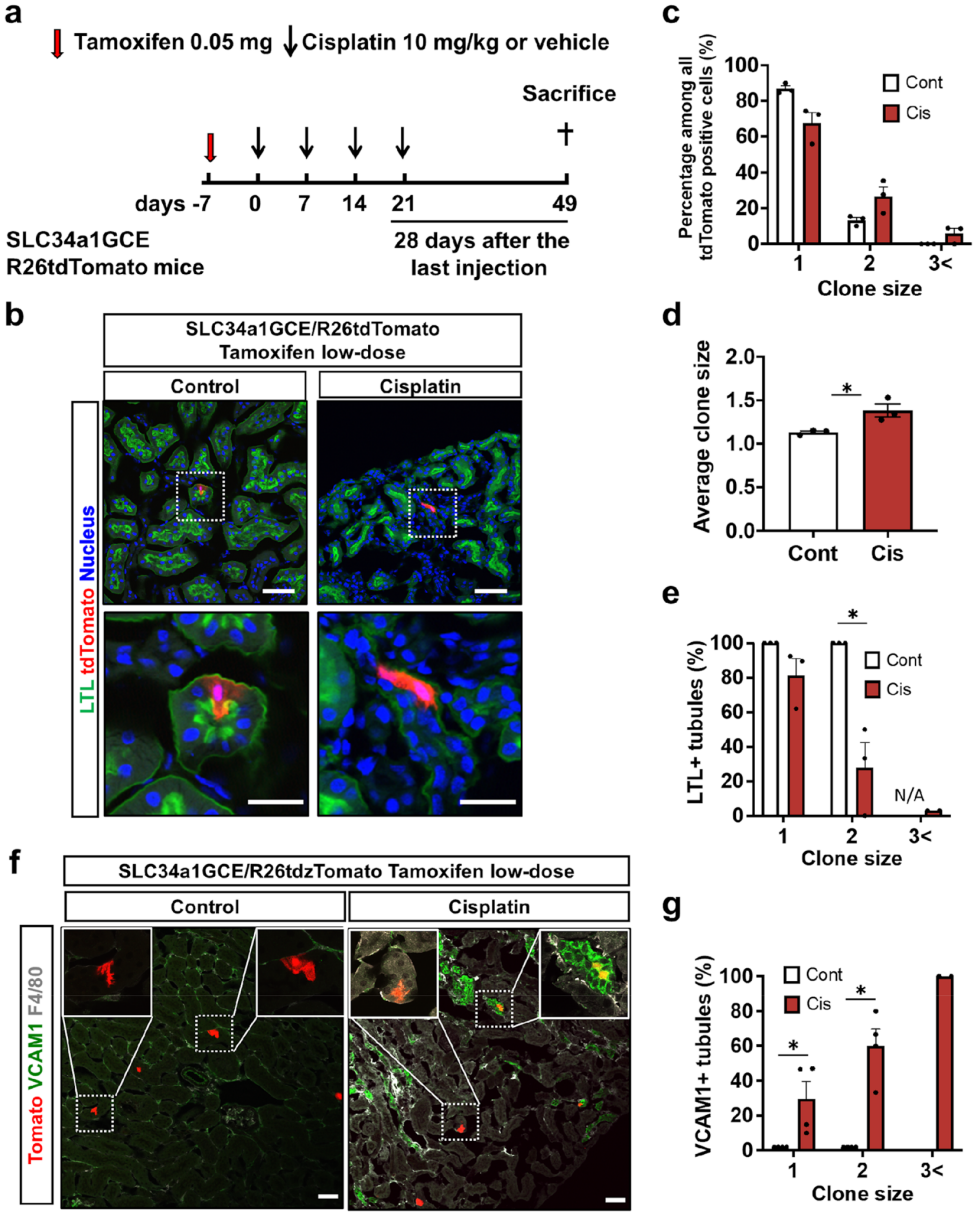
Clonal analysis of proximal tubular epithelial cells after repeated administration of 10 mg/kg of cisplatin. (**a**) Experimental scheme. Repeated administration of 10 mg/kg of cisplatin to the SLC34a1GCE R26tdTomato mice after labeling single proximal tubular epithelial cells by low-dose tamoxifen injection. (**b**) Representative images of tdTomato and immunostaining of LTL. Expansion of single tdTomato + clones in the cisplatin group. (**c**, **d**). Quantification of the clone size and its frequency. (**e**) Quantification of LTL + tubules by each clone size. (**f**) Representative images of tdTomato, and immunostaining of vcam-1 and F4/80. (**g**) Quantification of vcam-1 + tubules by each clone size. Data are the mean ± SE. *P < 0.05. Scale bar = 50 μm in LPF and 20 μm in HPF.

Using our bigenic mice, we can label a single proximal tubular cell by low-dose tamoxifen injection^29,31^ and the consecutively labeled cell number indicates the number of divisions of the labeled cells during the observation period. As tdTomato + tubular epithelia were solely responsible for the tubular regeneration, we then performed in vivo clonal analysis to directly demonstrate the relationship between tubular epithelial proliferation during repeated cisplatin-induced injury and tubular maladaptive repair in the chronic phase. In this analysis, only sparsely and randomly labeled tubular epithelial cells were included in the subsequent analysis. After labeling single proximal tubular epithelial cells by low-dose tamoxifen injection, mice were administered 10 mg/kg of cisplatin or saline once a week for four weeks and then euthanized at 28 days after the last cisplatin injection (Fig. 6a). After repeated kidney injury, tdTomato + clones expanded, confirming active epithelial cell proliferation during the repair process (Fig. 6b). The number of larger sized clones was higher and the average clone size was larger in the kidneys of the mice receiving cisplatin (Fig. 6c,d). Of note, tubules containing larger clones were more likely to be negative for LTL (Fig. 6b,e). Tubules containing larger clones were also often positive for vcam1 surrounded by F4/80 + macrophages, whereas tubules containing a single clone were often negative for vcam1 (Fig. 6f,g). This suggested that proliferation of tubular epithelia after repeated cisplatin injection is not sufficient for complete repair and instead leads to the proinflammatory failed-repair tubular state.

## Discussion

In this study, we demonstrated the mechanisms of repeated cisplatin-induced kidney injury focusing on DNA damage caused by each cisplatin injection. In this model, multiple injections of 10 mg/kg of cisplatin in seven-day intervals induced more severe renal tubular injury accompanying DNA damage than a single-dose injection, whereas 6 mg/kg of cisplatin in a seven-day interval or 10 mg/kg of cisplatin in a 14-day interval did not. Proximal tubular epithelia undergoing repair were still entering the cell cycle after receiving 10 mg/kg of cisplatin, thereby facilitating additional DNA damage induced by the next dose cisplatin, which exacerbated the kidney damage. In addition, clonal analysis at a later timepoint after injury using solely labeled proximal tubular cells demonstrated that bulk of tubular epithelia entering the cell cycle and cell division during repeated cisplatin-induced injury failed to repair and remained in a proinflammatory state.

Recently reported mouse models confirmed that repeated low-dose cisplatin administration induces kidney injury and the subsequent development of CKD^22–25^. These models enabled us to investigate the cisplatin-induced AKI to CKD transition observed in clinical practice. However, most studies evaluated kidneys only at the final point when the irreversible CKD phenotype was completed. In order to elucidate the mechanisms underlying the cisplatin-induced AKI to CKD transition, examination during multiple administrations is desirable; however, few studies have done this. To our knowledge, only one study focused on the responsible mechanisms in these repeated cisplatin models by comparing the kidneys of mice receiving two doses of cisplatin with those receiving a single dose^27^. They revealed increased proximal tubular damage in mice receiving two doses of 15 mg/kg cisplatin in a two-week interval compared with those receiving a single dose. In addition, their pathway analysis using microarray data confirmed the cell death pathway as a key determinant that leads to chronic kidney disease after repeated cisplatin-induced kidney injury.

Renal proximal tubular DNA damage is one of the most important initial responses after cisplatin administration. In response to milder DNA damage, DNA is repaired and cell death is prevented, but when the DNA damage is severe and beyond the reparative capacity, it initiates the activation of apoptotic pathways to induce tubular cell death and subsequent tubular injury^34^. In the present study, we expanded the previous study by administering cisplatin in different frequencies, doses, and intervals, and focused on the DNA damage upstream of the cell death pathway. As a result, we demonstrated that the dose, frequency, and interval of cisplatin administration determines the severity of DNA damage, and robust DNA damage was observed when higher doses of cisplatin were repeatedly administered in a shorter interval. DNA damage includes mismatched base pairs, base loss, SSB, and DSB^35^. DSB are considered the most hazardous type of DNA damage^36^. Based on our comet study, the frequency of DSB as well as SSB increased.

In general, cisplatin-induced DNA damage, including anti-tumor effects and adverse side effects, is marked, especially in rapidly proliferating cells^37^. Although normal quiescent renal proximal tubular cells have low rates of cell proliferation, in response to tubular injury and cell death, the surviving tubular epithelial cells enter the cell cycle and rapidly proliferate^17,38^. Immunostaining of Ki-67 revealed that tubular regeneration is incomplete and cells are still entering the cell cycle seven days after cisplatin administration at 10 mg. Considering these observations, multiple doses of cisplatin without a sufficient interval may be more damaging because rapidly proliferating cells that are susceptible to cisplatin-induced DNA damage remain.

By tracing a single tubular epithelial cell using lineage tracing analysis, we were able to assess the direct relationship between cell proliferation after cell cycle reentry and subsequent maladaptive repair. Clonal analysis using solely labeled proximal tubular cells revealed that the clone size increased through repeated injury, but regenerative tubules failed to repair and remained in a proinflammatory state. Active proliferation of tubular epithelia after repeated cisplatin induced-kidney injury did not necessarily lead to complete repair, and may have promoted kidney injury through more cumulative DNA damage. This is consistent with our previous finding that active proliferation of the tubular epithelia after kidney injury induced by IRI is not always beneficial for complete repair after kidney injury^31^. In addition, the regenerative proximal tubules which had proliferated actively tended to express vcam1 in the chronic phase after injury. Based on the recent single cell-based analysis of transcriptome using IRI kidney^33^, vcam1 was a sensitive marker for proinflammatory failed-repair tubules in our repeated cisplatin-induced kidney injury mouse model. To our knowledge, this is the first report that vcam1 is expressed in renal proximal tubules, and not in the endothelium, after repeated cisplatin-induced kidney injury and these vcam1 + tubules induced sustained inflammation through F4/80 + macrophages recruitment. CCL2 has been reported to be one of the major pro-inflammatory chemokines secreted by vcam1 + failed-repair tubules that recruit monocytes^33^ and has also reported to be a useful marker of progressive kidney injury in four doses of weekly cisplatin injection^39^. Our results revealed that CCL2 expression correlated well with cisplatin-induced kidney injury after four cisplatin injection where many failed-repair tubules were observed, but did not after single or dual injection. These results confirm previously reported findings.

Our study has several potential limitations. First, although we demonstrated that additional cisplatin injection before tubular repair is completed exacerbates kidney injury through DNA damage for up to two doses, other factors, such as inflammation or profibrogenic signaling, might have played a role after the third dose of cisplatin. Second, specific clinical markers for preventing kidney injury remain unclear and future experiments are needed. Third, this model is different from the representative patients receiving cisplatin who are usually elderly with comorbidities including cancer. Therefore, the effects of such comorbidities on this model remain unclear and future experiments are required. Lastly, as the efficiency of recombination in SLC34a1GCE mice in the outer medullary proximal tubules where severe injury occurs by cisplatin was low, we were unable to perform clonal analysis in this region.

In conclusion, in the repeated low-dose cisplatin-induced chronic kidney injury mouse model, proximal tubular epithelia enter the cell cycle and actively proliferate after each cisplatin injection, thereby increasing the amount of additional DNA damage induced by the next dose, which leads to chronic irreparable kidney disease. However, systemically promoting DNA damage repair is not an ideal renoprotective strategy, because cisplatin exerts anticancer effect through DNA damage and treatments for cisplatin nephropathy should not be at the expense of the antitumor effects of the cisplatin. In order to continue cisplatin treatment for cancer patients, due to the lack of definitive treatments for cisplatin nephropathy, appropriate setting of the dosage and intervals according to renal function, and careful monitoring of renal function or biomarkers are required.

## Supplementary Information

Supplementary Information.
