# Supplementary material for: Cumulative DNA damage by repeated low-dose cisplatin injection promotes the transition of acute to chronic kidney injury in mice
Source: Sci Rep. 2021 Oct 22;11:20920. doi: 10.1038/s41598-021-00392-6 (PMC8536734; doi:10.1038/s41598-021-00392-6)
Supplement: Supplementary file 1 — Supplementary Information. [file 41598_2021_392_MOESM1_ESM.docx]

Supplementary Information

**Cumulative DNA damage by repeated low-dose cisplatin injection promotes the transition of acute to chronic kidney injury in mice**

Noriyuki Yamashita, Kunihiro Nakai, Tomohiro Nakata, Itaru Nakamura,

Yuhei Kirita, Satoaki Matoba, Benjamin D. Humphreys, Keiichi Tamagaki,

and Tetsuro Kusaba

**Figure S1. qPCR analysis of kidneys receiving cisplatin at 7 days or 14 days after injection.**

Quantitative PCR from whole kidneys for the representative markers of mature tubules (*Lrp2*), inflammation (*Tnfa, Adgre1, and Ccl2*), and myofibroblast activation (*Acta2*).

Data are the mean ± SE. * p < 0.05, † p < 0.05 vs other groups.

**
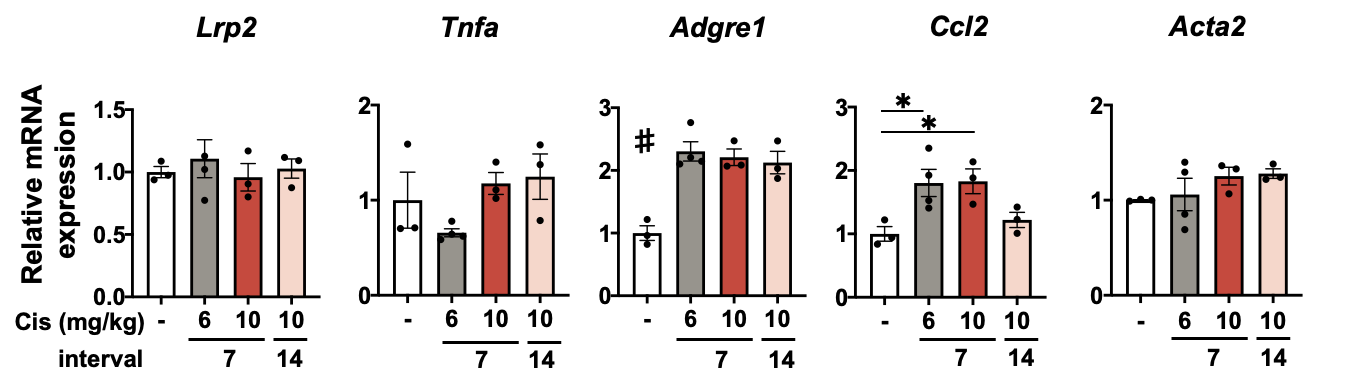
**

**Figure S2. qPCR analysis of kidneys receiving a single or double cisplatin.**

Quantitative PCR from whole kidneys for the genes related to DNA repair.

Data are the mean ± SE. * p < 0.05, † p < 0.05 vs other groups.


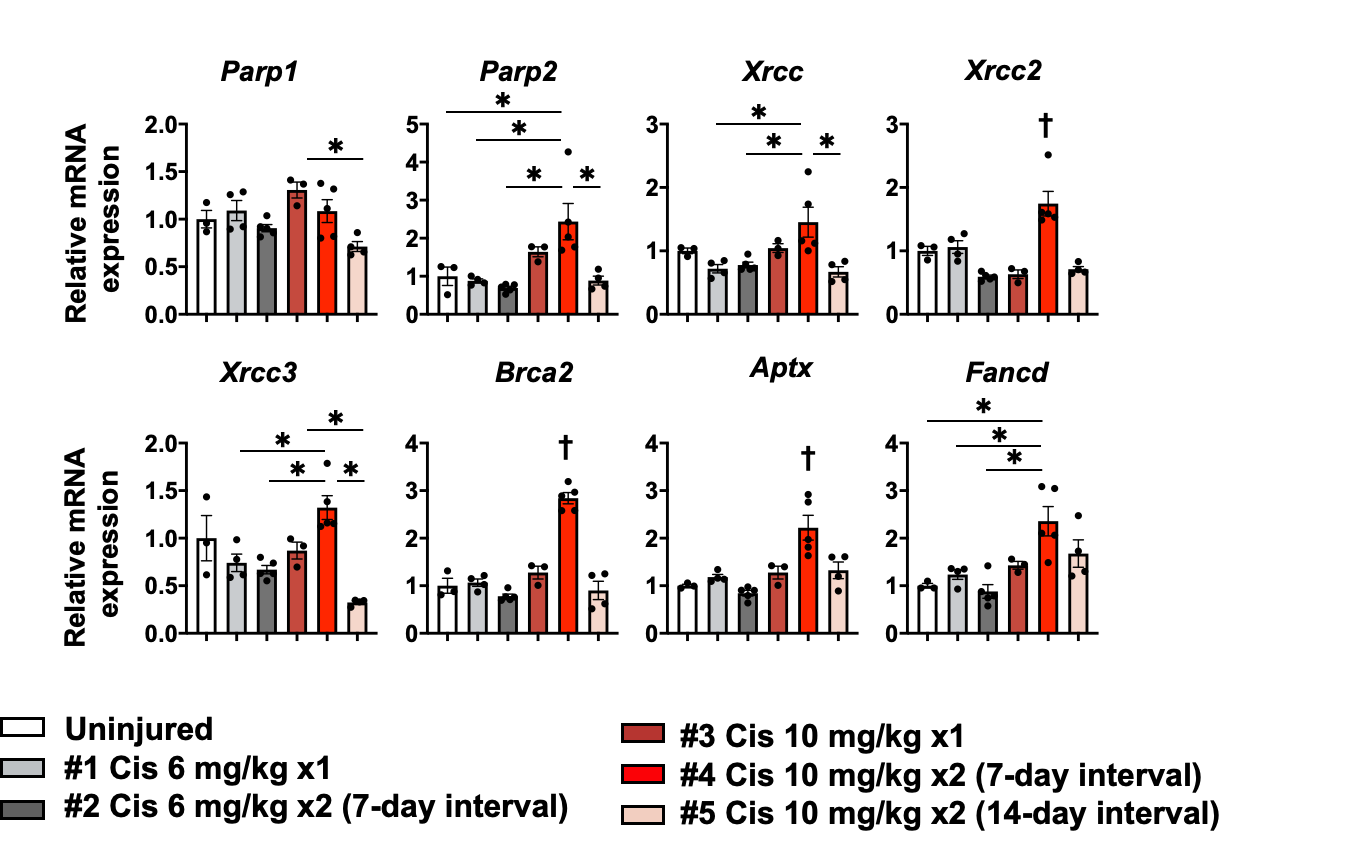


**Figure S3. qPCR analysis of kidneys at later phase after repeated cisplatin injection.**

Quantitative PCR from whole kidneys for the representative markers of mature tubules (*Lrp2*), kidney injury (*Havcr1*), inflammation (*Ccl2*), and fibrosis (*Col1a1* and *Tgfb1*).

Data are the mean ± SE. * p < 0.05.


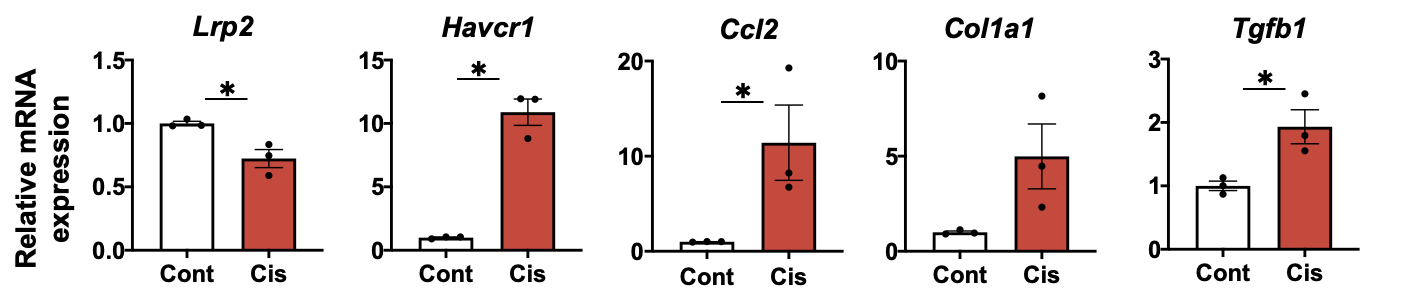


**Figure S4. Immunostaining of VCAM-1 and F4/80.**

Vcam-1+ failed-repair proximal tubules were surrounded by F4/80+ macrophages in the cisplatin model.


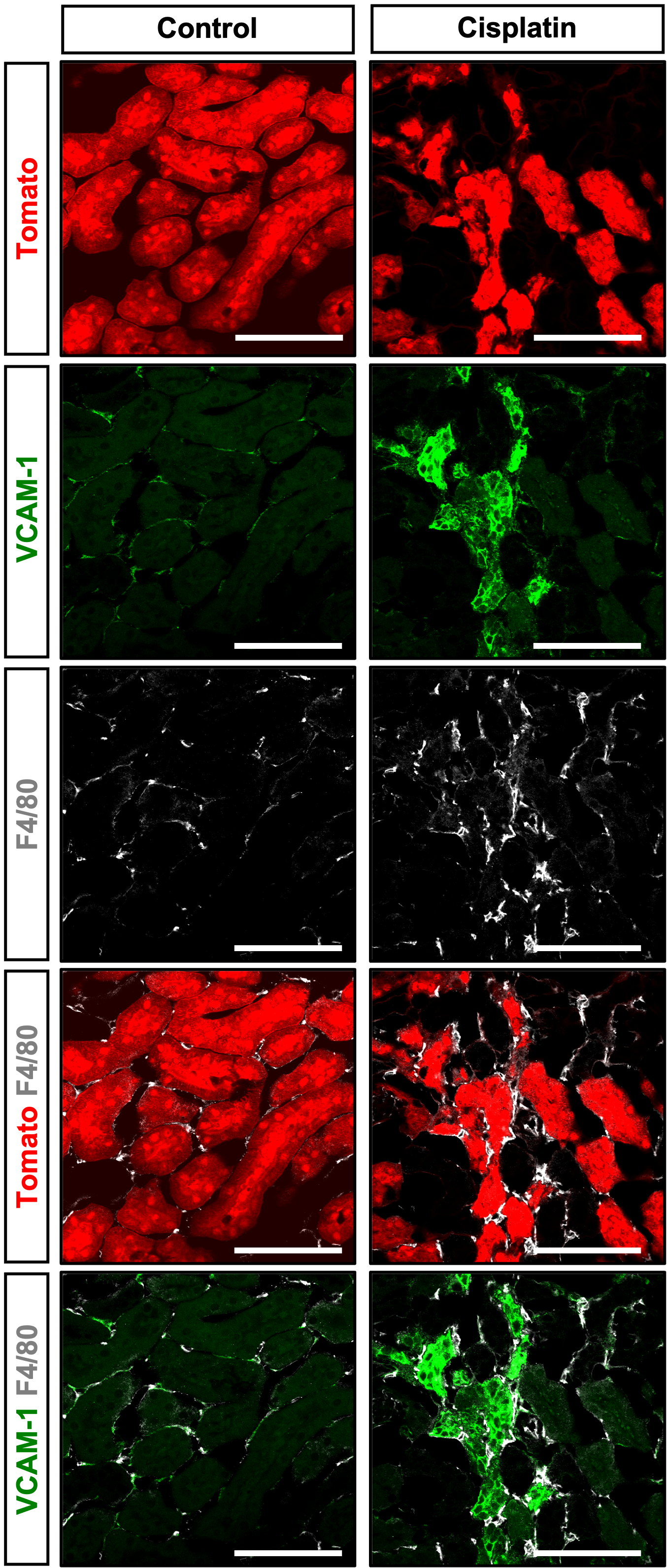


**Supplementary Table 1**. The primary and secondary antibodies for immunostaining

|  | Source | Catalog # | Vendor |
| --- | --- | --- | --- |
| **Primary antibody** |  |  |  |
| KIM-1 | Goat | AF1817 | R&D |
| Megalin | Goat | sc-16478 | SantaCruz |
| γH2AX | Rabbit | 2577S | CST |
| Ki67 | Rabbit | NB110-89719 | Novus |
| PDGFRβ | Rabbit | NBP1-47232 | Novus |
| FITC-conjugated LTL |  | FL1321 | Vector Labs |
| Laminin | Rabbit | ab11575 | abcam |
| Vcam-1 | Rabbit | ab134047 | abcam |
| F4/80 | Rat | 123102 | Biolegend |
| **Dye conjugated**  **secondary antibody** |  |  |  |
| anti-rabbit antibody | Goat | A11008 | Thermo Fisher Scientific |
| anti-rat antibody | Goat | A11006 | Thermo Fisher Scientific |
| anti-rat antibody | Goat | A21247 | Thermo Fisher Scientific |
| **HRP conjugated**  **secondary antibody** |  |  |  |
| anti-rabbit antibody | Goat | ab80437 | abcam |
| anti-rat antibody | Goat | ab7097 | abcam |
| anti-goat antibody | Rabbit | Ab2768 | SantaCruz |

**Supplementary Table 2**. The primers for qPCR

| Gene | Forward | Reverse |
| --- | --- | --- |
| *Lrp2* | AAAATGGAAACGGGGTGACTT | GGCTGCATACATTGGGTTTTCA |
| *Col1a1* | TAAGGGTCCCCAATGGTGAGA | GGGTCCCTCGACTCCTACAT |
| *Tgfb1* | GAACCCCCATTGCTGTCCC | AGCCCTGTATTCCGTCTCCT |
| *Tnfa* | AAGGCTGCCCCGACTACG | AGGTTGACTTTCTCCTGGTATGAG |
| *Adgre1* | TTCTGCTGTGGAAATGCAAG | AGAGGGGCTGGTAGGTTGAT |
| *P53* | ATAGAGACGCTGAGTCCGGTTC | CCTAAGCCCAAGAGGAAACAGA |
| *P21* | GTGGGTCTGACTCCAGCCC | CCTTCTCGTGAGACGCTTAC |
| *Bax* | CCGGCGAATTGGAGATGAACT | CCAGCCCATGATGGTTCTGAT |
| *Bcl-2* | GCTACCGTCGTGACTTCGC | CCCCACCGAACTCAAAGAAGG |
| *Pcna* | TTGCACGTATATGCCGAGACC | GGTGAACAGGCTCATTCATCTCT |
| *Fen-1* | ACCAGTTCCTGATTGCTGTTC | TCATGCGGATGGTACGGTAGA |
| *Cdk1* | AGGTACTTACGGTGTGGTGTAT | CTCGCTTTCAAGTCTGATCTTCT |
| *Top2a* | TGCTCCGCCCAGATACCTAC | TGGGTCCCTTTGTTTGTTATCAG |
| *Ccl2* | GCTCAGCCAGATGCAGTTAA | TCTTGAGCTTGGTGACAAAAACT |
| *Acta2* | GTCCCAGACATCAGGGAGTAA | TCGGATACTTCAGCGTCAGGA |
| *Parp1* | GTGACTTTTTAGCGGAGTACGC | CCAGCGGTCAATCATACCCAG |
| *Parp2* | CAGCACGCAGGATGAAAGTAA | CTGGTAACCGGCCTTGATTTG |
| *Xrcc1* | TCCACCCTCAAGAGACCCAAA | AGCGGAAGGGGTTCTGGAA |
| *Xrcc2* | TACCACTTTGACATGCTACGGC | CACTGACTCGGTCTATCCAGTA |
| *Xrcc3* | CGAATTACTGCTGCGGTTAAGA | CCCGAAGGTGTAGAGAGGCA |
| *Brca2* | ATGCCCGTTGAATACAAAAGGA | ACCGTGGGGCTTATACTCAGA |
| *Aptx* | TGCTGGTTGGTGAGACAGGA | ACTCTGCTTTCAACTGTACTTGC |
| *Fancd2* | TAATGGCCTGGAGTCCTACAC | CTCTTGGAGTAAAATGTGCCCA |
| *Vcam1* | CCGGCATATACGAGTGTGAA | GATGCGCAGTAGAGTGCAAG |
| *Havcr1* | AAACCAGAGATTCCCACACG | GTCGTGGGTCTTCCTGTAGC |
| *Gapdh* | TGCGACTTCAACAGCAACTC | CTTGCTCAGTGTCCTTGCTG |
